# Effective treatment of cutaneous and subcutaneous malignant tumours by electrochemotherapy.

**DOI:** 10.1038/bjc.1998.388

**Published:** 1998-06

**Authors:** L. M. Mir, L. F. Glass, G. Sersa, J. TeissiÃ©, C. Domenge, D. Miklavcic, M. J. Jaroszeski, S. Orlowski, D. S. Reintgen, Z. Rudolf, M. Belehradek, R. Gilbert, M. P. Rols, J. Belehradek, J. M. Bachaud, R. DeConti, B. Stabuc, M. Cemazar, P. Coninx, R. Heller

**Affiliations:** LPPMB/Biochimie-Enzymologie, URA 147 CNRS, Institute Gustave-Roussy, Villejuif, France.

## Abstract

Electrochemotherapy (ECT) enhances the effectiveness of chemotherapeutic agents by administering the drug in combination with short intense electric pulses. ECT is effective because electric pulses permeabilize tumour cell membranes and allow non-permeant drugs, such as bleomycin, to enter the cells. The aim of this study was to demonstrate the anti-tumour effectiveness of ECT with bleomycin on cutaneous and subcutaneous tumours. This article summarizes results obtained in independent clinical trials performed by five cancer centres. A total of 291 cutaneous or subcutaneous tumours of basal cell carcinoma (32), malignant melanoma (142), adenocarcinoma (30) and head and neck squamous cell carcinoma (87) were treated in 50 patients. Short and intense electric pulses were applied to tumours percutaneously after intravenous or intratumour administration of bleomycin. The tumours were measured and the response to the treatment evaluated 30 days after the treatment. Objective responses were obtained in 233 (85.3%) of the 273 evaluable tumours that were treated with ECT. Clinical complete responses were achieved in 154 (56.4%) tumours, and partial responses were observed in 79 (28.9%) tumours. The application of electric pulses to the patients was safe and well tolerated. An instantaneous contraction of the underlying muscles was noticed. Minimal adverse side-effects were observed. ECT was shown to be an effective local treatment. ECT was effective regardless of the histological type of the tumour. Therefore, ECT offers an approach to the treatment of cutaneous and subcutaneous tumours in patients with minimal adverse side-effects and with a high response rate.


British Journal of Cancer (1998) 77(12), 2336–2342
© 1998 Cancer Research Campaign

### Effective treatment of cutaneous and subcutaneous malignant tumours by electrochemotherapy

**LM Mir[1], LF Glass[2,3], G Serša[4], J Teissié[5], C Domenge[6], D Miklavčič[7], MJ Jaroszeski[3,8], S Orlowski[9], DS Reintgen[3,8], Z Rudolf[4], M Belehradek[6], R Gilbert[10], M-P Rols[5], J Belehradek Jr[1], JM Bachaud[11], R DeConti[2,3], B Štabuc[4], M Čemažar[4], P Coninx[12] and R Heller[3,8]**

[1]LPPMB/Biochimie-Enzymologie, URA 147 CNRS, Institute Gustave-Roussy, F-94805 Villejuif, France; Department of [2]Internal Medicine, University of South Florida, Tampa, FL 33612, USA; [3]Cutaneous Oncology Program, H Lee Moffitt Cancer Center and Research Institute, FL 33612, Tampa, USA; [4]Institute of Oncology, SI-1105 Ljubljana, Slovenia; [5]LPTF, UPR 8221 CNRS, F-31062 Toulouse, France; [6]Department of Head and Neck Carcinology, Institute Gustave-Roussy, F-94805 Villejuif, France; [7]University of Ljubljana, Faculty of Electrical Engineering SI-1000 Ljubljana, Slovenia; [8]Department of Surgery, University of South Florida, FL 33612, Tampa, USA; [9]SBPM/DBCM CEA, URA 2096 CNRS, CEN Saclay, F-91191 Gif/Yvette, France; [10]Department of Chemical Engineering, University of South Florida, FL 33612 Tampa, USA; [11]Department of Radiotherapy, Centre Claudius-Régaud, F-31052 Toulouse, France; [12]Department of Head and Neck Carcinology, Institut Jean-Godinot, F-51056 Reims, France

**Summary** Electrochemotherapy (ECT) enhances the effectiveness of chemotherapeutic agents by administering the drug in combination with short intense electric pulses. ECT is effective because electric pulses permeabilize tumour cell membranes and allow non-permeant drugs, such as bleomycin, to enter the cells. The aim of this study was to demonstrate the anti-tumour effectiveness of ECT with bleomycin on cutaneous and subcutaneous tumours. This article summarizes results obtained in independent clinical trials performed by five cancer centres. A total of 291 cutaneous or subcutaneous tumours of basal cell carcinoma (32), malignant melanoma (142), adenocarcinoma (30) and head and neck squamous cell carcinoma (87) were treated in 50 patients. Short and intense electric pulses were applied to tumours percutaneously after intravenous or intratumour administration of bleomycin. The tumours were measured and the response to the treatment evaluated 30 days after the treatment. Objective responses were obtained in 233 (85.3%) of the 273 evaluable tumours that were treated with ECT. Clinical complete responses were achieved in 154 (56.4%) tumours, and partial responses were observed in 79 (28.9%) tumours. The application of electric pulses to the patients was safe and well tolerated. An instantaneous contraction of the underlying muscles was noticed. Minimal adverse side-effects were observed. ECT was shown to be an effective local treatment. ECT was effective regardless of the histological type of the tumour. Therefore, ECT offers an approach to the treatment of cutaneous and subcutaneous tumours in patients with minimal adverse side-effects and with a high response rate.

**Keywords:** electrochemotherapy; clinical trial; basal cell carcinoma; malignant melanoma; adenocarcinoma; head and neck squamous cell carcinoma

Chemotherapy is a widely used treatment for a broad range of cancers. In many instances, the response rate is low. In melanoma, for example, partial response rates range from 20% to 45%, with complete responses of less than 5% (Buzaid and Murren, 1992; Coates, 1992; Nathanson and Jilani, 1993; Yeung, 1994). One possible reason for this low response rate is the difficulty of some drugs to cross the cell membrane and reach their intracellular site of action. The application of short and intense electric pulses can reversibly permeabilize membranes of all living cells, including mammalian, bacterial, yeast and plant cells (Mir et al, 1988; Neumann et al, 1989; Rols and Teissié, 1990; Orlowski and Mir, 1993). These electric pulses have been used in vitro to introduce drugs, foreign DNA and other exogenous molecules into cells (Orlowski and Mir, 1993).

Bleomycin is a very potent cytotoxic molecule when introduced inside the cell. A few hundred molecules are sufficient to be cytotoxic (Poddevin et al, 1991; Tounekti et al, 1993). However, bleomycin does not freely diffuse through the plasma membrane and has very limited access to the cytosol (Orlowski et al, 1988; Poddevin et al, 1991). Bleomycin normally enters cells through interaction with a membrane protein that mediates its internalization (Pron et al, 1993). Response is a function of the presence or absence of this membrane protein (Pron et al, 1993; 1994). This could be a significant reason why bleomycin has had limited success as an anti-tumour agent. Electropermeabilization of cells as well as tissues allows bleomycin to enter the cytosol directly and to exert fully its cytotoxic potential (Orlowski et al, 1988; Poddevin et al, 1991; Tounekti et al, 1993; Belehradek et al, 1994). Therefore, bleomycin is an excellent candidate for combining with electric pulses because it is non-permeant and at the same time highly cytotoxic once inside the cell.

The anti-tumour effectiveness of bleomycin was shown to be greatly increased by the local delivery of permeabilizing electric pulses at the tumour in preclinical trials with mice (Belehradek et al, 1991; Mir et al, 1991). The permeabilizing effect is restricted to an area encompassed by the electrodes. Electric pulses delivered after the administration of bleomycin actually increased drug delivery to tumours (Belehradek et al, 1994). However, electric pulses delivered alone did not elicit an anti-tumour response. Anti-tumour effects

*Received 11 August 1997*
*Revised 5 November 1997*
*Accepted 12 November 1997*

*Correspondence to:* LM Mir, URA 147 CNRS, Institute Gustave-Roussy, 39 rue Camille Desmoulins, F-94805 Villejuif Cedex – France were observed in many different animal models (Mir et al, 1991; Belehradek et al, 1991; Salford et al, 1993; Mir, 1994; Serša et al, 1994; 1995; Yamaguchi et al, 1994; Čemažar et al, 1995; Heller et al, 1995). Anti-tumour treatment with this combined therapy can be used in tumour systems in which bleomycin alone was not typically used (Mir et al, 1996). This new anti-tumour therapy that combines the administration of a non-permeant drug such as bleomycin with local permeabilizing electric pulses was termed electrochemotherapy (ECT) (Mir et al, 1991; Mir, 1994). The principle of ECT is characterized by a more efficient manner of drug delivery that increases the effectiveness of the administered drug with reduced side-effects.

The first ECT clinical trial performed in Villejuif, France, treated cutaneous nodules of metastatic squamous cell carcinoma present on the head and neck (Belehradek et al, 1993). Objective responses were obtained in 72% of 40 treated nodules in eight patients. The therapy was well tolerated by the patients. Preliminary sedation 1 h before ECT or in one case short neurolept-analgesia was used. Subsequently, several clinical trials have been initiated in other cancer centres. Preliminary reports of these trials on 15 patients have been published (Heller, 1995; 1996; Rudolf et al, 1995; Domenge et al, 1996). Patients with basal cell carcinoma, malignant melanoma, adenocarcinoma or squamous cell carcinoma were enrolled in these trials to determine if ECT is applicable to tumours of other histological types. All trials used bleomycin, which was administered several minutes before delivery of electric pulses. This report presents a summary of results obtained from clinical trials performed independently in five cancer centres and included the treatment of 291 tumours in 50 patients.

### PATIENTS AND METHODS

### Patients and inclusion criteria

Ten patients with basal cell carcinoma (BCC), 20 with metastatic malignant melanoma, three with adenocarcinoma of the salivary gland or breast and 17 with head and neck squamous cell carcinoma (HNSCC) were entered in trials initiated in five different cancer centres. These were located in Villejuif (VI), Toulouse (TO), Reims (RE), France; Ljubljana (LJ), Slovenia; and Tampa (TA), United States. A total of 32 primary BCC tumours, 142 metastatic malignant melanoma deposits, 30 adenocarcinoma subcutaneous metastases and 87 metastatic HNSCC nodules were treated in 66 sessions. At the time of their inclusion in ECT trials, patients with primary BCC of the skin had previous surgery or multiple lesions and refused additional conventional therapies, such as surgery. Patients with metastatic malignant melanoma, adenocarcinoma and HNSCC presented with recurrent lesions after several previous treatments by surgery, radiotherapy and/or chemotherapy. Of the total patient population, only 2 of the 50 (both malignant melanoma) had received bleomycin before ECT. Signed informed consent was obtained from all patients. Trials were performed after approval of the corresponding ethics and institutional review committees.

### Protocols for bleomycin administration

Bleomycin was injected intravenously (i.v.), in a rapid bolus (30 s duration) at least 3 min before electric pulse delivery (VI, TO, RE and LJ). The bleomycin dose was either 18 units $m^{-2}$ (10 mg $m^{-2}$) or 27 units $m^{-2}$ (15 mg $m^{-2}$). Alternatively, bleomycin was injected either i.v., 10 units $m^{-2}$ (5.6 mg $m^{-2}$) at an infusion rate of 1.5 units $min^{-1}$, or intratumorally (i.t.), 0.25 to 1.0 units per treated tumour in less than 30 s at least 10 min before electric pulse delivery (TA).

### Protocols for electric pulse delivery

Treatment included applying rectangular-wave electric pulses directly to tumours after bleomycin administration. The French and Slovenian centres (VI, RE, TO and LJ) used electrodes that consisted of two stainless steel strips 10 mm wide and 0.6 mm thick. Insulating material was used to maintain a fixed gap between the two strips. Spacing between the electrodes was 7 mm for VI, RE and LJ and 6 mm for TO. Electric pulses were delivered by a PS 15 electropulsator (Jouan, Nantes, France) that was designed to obey the electric current limits set by the European Community (*Commission for Industrial Electricity*, 1984). The US centre (TA) used electrodes consisting of two stainless steel squares of 20 mm mounted on a vernier caliper or a circular array of six needles. The caliper-mounted electrodes allowed the gap to be adjusted according to the size of the tumour and the needle array electrode was fixed at a 10 mm diameter. Electric pulses were delivered by a BTX T820 generator (Genetronics, San Diego, CA, USA).

**Table 1** Patient information and response to treatment

| Histological type | Mean patient age (range) | Mean tumour diameter (mm) (range) | Response to electrochemotherapy |
|---|---|---|---|
| Basal cell carcinoma | 55.8 | 7.7 | 100% OR |
| 10 patients, 32 tumours | (38–71) | (4–14) | (75% CR, 25% PR) |
| Melanoma | 55.5 | 8.1 | 92.2% OR |
| 20 patients, 142 tumours | (33–76) | (2–52) | (52.8% CR, 39.4% PR) |
| Adenocarcinoma | 54.3 | 8.5 | 100% OR[a] |
| 3 patients, 30 tumours | (39–67) | (3–17) | (100% CR) |
| Squamous cell carcinoma | 53.0 | 17.5 | 62.3% OR[b] |
| 17 patients, 87 tumours | (37–67) | (3–125) | (42.8% CR, 19.5% PR) |
| Total | 54.6 | 11.24 | 85.3% OR[c] |
| 50 patients, 291 tumours | (37–76) | (2–125) | (56.4% CR, 28.9% PR) |

OR, objective responses; CR, complete responses; PR, partial responses. [a]Based on 22 evaluable tumours; [b]based on 77 evaluable tumours; [c]based on 273 evaluable tumours.

© Cancer Research Campaign 1998

**Table 2** Responses of basal cell carcinoma tumours treated with electrochemotherapy

| Patient | Bleomycin dose (units)[a] | Electric pulses[b] | Number of tumours | Response |
|---|---|---|---|---|
| TA4 | i.v./10 | 8 | 4 | 1 CR, 3 PR |
| TA5 | i.v./10 | 8 | 2 | 2 PR |
| TA7 | i.t./2.25 | 8 | 3 | 3 CR |
| TA8[c] | i.t./2.75/5.5/4 | 8/6/6 | 3/2/5 | 7 CR, 3 PR |
| TA9 | i.t./1.75 | 8 | 2 | 2 CR |
| TA11 | i.t./4 | 8 | 4 | 4 CR |
| TA13 | i.t./0.5 | 8 | 1 | 1 CR |
| TA16 | i.t./2.0 | 6 | 2 | 2 CR |
| TA17 | i.t./2.0 | 6 | 2 | 2 CR |
| TA19 | i.t./1.25 | 6 | 2 | 2 CR |

[a]Bleomycin dose and route of administration; i.v., intravenous; i.t., intratumour. [b]Electric pulses – number of electric pulses administered to each treated tumour. [c]Patient treated in three sessions.

**Table 3** Responses of melanoma tumours treated with electrochemotherapy

| Patient | Bleomycin dose (units)[a] | Electric pulses[b] | Number of tumours | Response |
|---|---|---|---|---|
| TA1 | i.v./10 | 8 | 4 | 3 SD[c], 1 PD[c] |
| TA2 | i.v./10 | 8 | 4 | 3 CR, 1 SD |
| TA3 | i.v./10 | 8 | 2 | 2 PR |
| TA10[d] | i.t./1.5/3.0 | 8/6 | 2/2 | 3 CR, 1 PR |
| TA12[d] | i.t./5.0/6.0 | 8/6 | 6/5 | 11 CR |
| TA14[d] | i.t./5.25/6.5 | 6/6 | 5/17 | 22 CR |
| TA15 | i.t./5.75 | 6 | 4 | 3 PR, 1 PD |
| TA18 | i.t./5.5 | 6 | 3 | 3 CR |
| TA20 | i.t./2.75 | 6 | 4 | 4 CR |
| LJ1[d] | i.v./18/18 | 4+4/4+4 | 3/3[e] | 3 CR |
| LJ2 | i.v./18 | 4+4 | 1 | 1 PR |
| LJ3 | i.v./18 | 4+4 | 1 | 1 SD[f] |
| LJ4 | i.v./18 | 4+4 | 2 | 2 CR |
| LJ5 | i.v./18 | 4+4 | 1 | 1 PR[f] |
| LJ6[g] | i.v./18/18/18 | 4+4/4+4/4+4 | 2/6/13 | 19 CR, 1 SD, 1 PD[c] |
| LJ7 | i.v./18 | 8 | 1 | 1 PR |
| TO2[d] | i.v./18/18 | 4/4 | 10/10[e] | 10 PR |
| TO3 | i.v./18 | 4 | 22 | 4 CR, 18 PR |
| TO4 | i.v./18 | 8 | 11 | 10 PR, 1 SD |
| TO5 | i.v./18 | 8 | 11 | 1 CR, 9 PR, 1 SD[f] |

[a]Bleomycin dose and route of administration; i.v., intravenous; i.t., intratumour. [b]Electric pulses – number of electric pulses administered to each treated tumour. [c]Tumours located in areas poorly perfused by blood as indicated in text. [d]Patient treated in two sessions. [e]Same tumours treated in two sessions. [f]Not the whole tumour area was treated. [g]Patient treated in three sessions.

**Table 4** Responses of adenocarcinoma tumours treated with electrochemotherapy

| Patient | Origin | Bleomycin dose (units)[a] | Electric pulses[b] | Number of tumours | Response |
|---|---|---|---|---|---|
| TA6 | Breast | i.v./10 | 8 | 2 | 2 CR |
| VI9 | Salivary gland | i.v./27 | 4 | 20 | 20 CR |
| VI15 | Breast | i.v./27[c] | 4 | 8 | 8 NE[d] |

[a]Bleomycin dose and route of administration; i.v., intravenous; i.t., intratumour. [b]Electric pulses – number of electric pulses administered to each treated tumour. [c]Owing to the time required to treat the large tumours, a supplement of bleomycin was administered. [d]Tumours were non-evaluable because of too short follow-up.

Either four, six or eight electric pulses were delivered per treatment site by placing electrodes on the skin adjacent to the tumour. Electric pulses were delivered at a rate of one pulse per second. All the patients treated in VI, TO, RE and LJ received either four or eight electric pulses. In LJ this was accomplished by delivering four pulses then rotating the electrodes 90° and administering four additional pulses. This is denoted as a 4+4 configuration (Čemažar et al, 1995). Patients treated in TA received either eight (caliper electrodes) or six (needle array electrode) electric pulses. The ratio of voltage to electrode distance was 1300 V $cm^{-1}$ for all teams,

© Cancer Research Campaign 1998

except when otherwise stated. Skin contact was ensured by means of electrocardiography paste and shaving when necessary. For the treatment of small tumours, electrodes were placed at each side of the tumour and one series of electric pulses was delivered. For the treatment of large tumours, sequential electrical treatments were delivered at adjacent positions in order to cover the entire tumour surface.

Before administration of electric pulses, patients received either local or systemic anaesthesia. patients at VI with a small number of treatment sites received only sedatives (lorazepan or levomepromazine). Patients at VI, RE or TO with large tumours or with numerous sites were treated under neuroleptanalgesia using midazolam and alfentanil or under general anaesthesia. Patients at LJ received lidocaine spray over the treated surface. Patients at TA received a peritumoural injection of 1–3 ml of 1% lidocaine per lesion.

### Follow-up

During ECT and some hours later, patients were carefully monitored for treatment side-effects. The first few patients in the trial (VI 1–7) and any patient treated under neuroleptanalgesia or complete anaesthesia (VI 8–15, TO and RE) remained in the hospital for 24 h. In the other cases, the procedure was performed on an outpatient basis. All of them were examined as outpatients at regular intervals after ECT. Tumour measurements were made using a vernier caliper, and documented with photographs. Response rates were based on the tumour volume by measuring the longest diameter ($a$) and the next longest diameter ($b$) perpendicular to $a$. The tumour volume ($V$) was calculated by the formula: $V = \pi ab^2/6$.

When the results were analysed on a per treated nodule basis, the number of objective responses (OR) was determined by adding the number of complete responses (CR, no palpable or measurable tumour detected for at least 30 days after treatment) and partial responses (PR, greater than 50% decrease in tumour volume for at least 30 days after treatment). Stable disease (SD) was defined as no growth but less than 50% reduction in tumour volume; and progressive disease (PD) was defined as continued growth. To determine the response rate per patient, the poorest response on the per treated nodule basis was taken into account.

### RESULTS

### Clinical response

A total of 50 patients were treated at five different cancer centres in 66 ECT sessions. This included a total of 291 tumours of various sizes and of various histological types (Table 1). Of the 291 sites treated, 273 were evaluable. Objective responses were found in 233 (85.3%) of the evaluable tumours of which 154 (56.4%) were CR and 79 (28.9%) were PR. On a per patient basis, 48 patients were evaluable. Objective responses were found in 31 (64%) of the evaluable patients of which 17 (35%) were CRs and 14 (29%) were PRs.

### Basal cell carcinoma

Among the 32 BCC primary tumours treated (ten patients), a 100% response rate was observed after ECT (Table 2). Of these, 24 (75%) disappeared within 1 month and did not recur during a mean follow-up of 15 months (range 6–27 months) and were designated CRs. The other eight (25%) were found to be PRs. On a per patient basis, we obtained seven CRs (70%) and three PRs (30%). The size of all 15 control BCCs treated with either bleomycin or electric pulses alone progressed during the period of observation (100% PD).

The first two BCC patients were treated with an i.v. dose of bleomycin. Only one of the six (16.7%) tumours treated in these patients was a CR and five were PRs. The number of CRs after i.v. administration of bleomycin was unacceptable for BCC. Therefore, the other eight BCC patients received bleomycin i.t. In these patients CRs were found in 23 of 26 tumours and three were

**Table 5** Responses of head and neck squamous cell carcinoma tumours treated with electrochemotherapy

| Patient | Bleomycin dose (units)[a] | Electric pulses[b] | Number of tumours | Response |
|---|---|---|---|---|
| VI1[c] | i.v./18/18 | 4/4 | 1/11 | 12 CR |
| VI2 | i.v./18 | 4 | 1 | 1 SD |
| VI3 | i.v./18 | 4 | 6 | 6 SD |
| VI4 | i.v./18 | 4 | 1 | 1 PR |
| VI5 | i.v./18 | 4 | 1 | 1 SD |
| VI6[c] | i.v./18/18 | 8/8 | 3/8 | 3 CR, 5 PR, 3 SD |
| VI7 | i.v./18 | 8 | 2 | 2 CR |
| VI8[d] | i.v./18/27/18 | 8/4[e]/4[e] | 1/3/4 | 6 CR, 1 SD, 1 PD[f] |
| VI10[d] | i.v./27/27/27 | 4/4/4 | 5/1/2 | 1 CR, 3 PR, 3 SD, 1 PD |
| VI11 | i.v./27 | 8 | 6 | 3 CR, 1 PR, 2 SD |
| VI12 | i.v./27 | 4[e] | 10 | 10 NE[g] |
| VI13 | i.v./27 | 4 | 3 | 1 PR, 2 SD |
| VI14[c] | i.v./27/27[h] | 4/4 | 2/2[i] | 2 PD |
| RE1 | i.v./27 | 4 | 2 | 1 CR, 1 PR |
| RE2 | i.v./27 | 4 | 2 | 2 PR |
| RE3 | i.v./27 | 4 | 6 | 2 SD, 4 PD |
| TO1 | i.v./18 | 4 | 6 | 5 CR, 1 PR |

[a]Bleomycin dose and route of administration; i.v., intravenous; i.t., intratumour. [b]Electric pulses – number of electric pulses administered to each treated tumour. [c]Patient treated in two sessions. [d]Patient treated in three sessions [e]Four electric pulses at a field strength of 1000 V cm$^{-1}$ were administered. [f]Tumour pulsed outside the therapeutical window after bleomycin injection. [g]Tumours were non-evaluable because of too short follow-up. [h]Bleomycin was administered intra-arterially. [i]Same tumours treated in two sessions.

© Cancer Research Campaign 1998 

PRs. Two of the PRs were retreated and CRs were obtained. Therefore, examining BCCs that received an i.t. dose of bleomycin and one or two treatments, CRs were obtained in 25 of 26 (96.2%) treated tumours.

### Melanoma

Objective responses were seen in 131 (92.2%) of the 142 metastatic malignant melanoma nodules (20 patients) treated (Table 3). CRs were seen in 75 (52.8%) of the treated tumours that rapidly disappeared within 1–2 weeks after ECT. No regrowth of these nodules was reported. PRs were seen in 56 (39.4%) of the treated sites. On a per patient basis, we obtained six CRs (30%) and seven PRs (35%). All 15 control nodules treated with bleomycin alone were observed to have PD.

Of the 11 (7.7%) metastatic melanoma nodules that did not respond to ECT, two nodules were situated in a way that the entire tumour could not be treated. A total of five other treated tumours in two patients were located in poorly perfused areas. These two patients received i.v. bleomycin, which could account for the lack of response.

### Adenocarcinoma

All 22 (100%) evaluable adenocarcinoma metastatic nodules (three patients) responded to ECT (Table 4). All of these were found to be CRs. Eight additional metastatic nodules were treated but could not be evaluated because of a too short follow-up period. The two control nodules receiving bleomycin alone had PD.

### Head and neck squamous cell carcinoma

Objective responses were seen in 48 (62.3%) of the 77 HNSCC evaluable nodules (17 patients) treated (Table 5). Of these nodules, 33 (42.8%) were in CR and 15 (19.5%) in PR. Only eight (10.3%) of the nodules were PDs. Ten additional nodules were not evaluated because of too short follow-up of the patient. On a per patient basis, we obtained two CRs (12%) and four PRs (24%). In the case of the HNSCC nodules, it was necessary to distinguish between nodules that could be encompassed with one treatment using transcutaneous electric pulses and the very large nodules treated with sequential electrical treatments. The treatment of the large nodules was important for the determination of some parameters and constraints of electrochemotherapy. For these large nodules, massive necrosis and reduction in the height of the nodule was observed but, as expected, the deepest parts were not completely affected by ECT, presumably because they were not crossed by an electric field of sufficient intensity.

### Tolerance during and after the treatment

No significant modification of haemodynamic or cardiological parameters was noticed during ECT. A contraction of muscles located beneath the site of treatment was observed. The contractions were instantaneous, disappearing immediately after the end of each electric pulse (1/10 000th of a second in length). Several procedures, either local or systemic, as described above, were used for relief of the sensations that accompanied the contractions. General anaesthesia seems more appropriate when large and/or multiple nodules are treated, whereas local anaesthesia would be sufficient for the treatment of a few small tumours.

Erythema and slight oedema at the site of the treated areas were the only noticeable symptoms observed transiently and remained less than 24 h. Transient marks from electrodes were also often visible after ECT. Superficial leuconecrosis was observed in the case of the large tumours for which the skin was already altered before ECT. For patients receiving only sedatives or local anaesthesia, some pain was involved during the procedure, which subsided immediately after the last pulse was delivered. All patients agreed that it was tolerable and they would undergo the procedure again. As a matter of fact, several patients returned for treatment of additional lesions. In addition, no delayed pain was reported by the patients. In several cases, the pain associated with the tumour was attenuated after ECT. No enhanced systemic bleomycin toxicity was observed.

### DISCUSSION

Results reported here from five cancer centres included the treatment of 273 evaluable tumours with an objective response rate of 85.3%. ECT was safe and well tolerated by the patients. The high rate of objective responses was obtained regardless of the histological type of the treated tumours. These results are encouraging for the future development of ECT and illustrate that ECT has the potential to be a potent anti-tumour treatment even when the treatment protocol is varied.

The high rate of objective responses obtained regardless of histological type is in agreement with the fundamental basis of ECT. Cell electropermeabilization is a universal phenomenon occurring in all types of living cells and results from the interaction of electric fields with a low conductive membrane composed of lipids and proteins (Neumann et al, 1989; Orlowski and Mir, 1993). Once inside the cell, interaction of bleomycin with DNA results in a chemical reaction that generates highly cytotoxic DNA double-strand breaks (Poddevin et al, 1991; Tounekti et al, 1993). Thus, all histological types of tumours should be sensitive to ECT.

Electric pulses have to be delivered during the period for which the interstitial bleomycin concentration in the tumour is sufficient. This period was determined in one of the clinical situations examined (HNSCC) to be from 8 to 28 min after bleomycin i.v. bolus injection. This time frame is perhaps longer if bleomycin is injected slowly (for example at a rate of 1.5 units $min^{-1}$). However, the concentration of bleomycin at the treatment site is probably lower. Although there is a defined window of opportunity for successful treatment when administering bleomycin i.v., this does not present a problem because the procedure for applying electric pulses is very rapid (4–8 s) and can be repeated many times inside the therapeutical window in the case of very large tumours or several tumours.

An alternative procedure to i.v. bleomycin administration is i.t. injection, which proved to be effective as well. Moreover, if tumour treatment is associated with a local approach for analgesia, then every tumour can be treated independently, and the whole ECT session can be split in as many partial sessions as required. The results found in these studies indicate that ECT is an effective treatment irrespective of the bleomycin administration route. Therefore, the treatment can be administered in a variety of ways depending on the clinical situation.

An important observation was that ECT was safe and resulted in minimal side-effects. In particular, no significant modification of the haemodynamic or cardiological parameters was noticed even when the treated tumours were located in the chest above the

© Cancer Research Campaign 1998

cardiac region. However, there is a contraction of the muscles located beneath the electrodes as the muscle cells are excited by the electric pulses. Many patients had disagreeable sensations associated with the delivery of electric pulses. This was short lived and disappeared immediately after pulsing. In addition, sometimes transient marks from the electrodes were visible.

Thus, at the present time, ECT can already be considered as an effective treatment, whatever the histological type of the tumour. ECT has been efficient on multiple BCCs in patients previously disfigured by surgery who refused further surgery. Scarring was minimal in the treated sites, which makes ECT an obvious advantage over a surgical excision. ECT was also effective as a local treatment for malignant melanoma with a 92.2% objective response. ECT has also proven anti-tumour effectiveness on otherwise untreatable HNSCC nodules as these nodules, resistant to conventional chemotherapy, were located in areas previously treated by surgery and radiotherapy. An important observation was that results were consistent among the different centres even though the procedure was varied. This highlights that the basic premise of ECT, permeabilization of tumour cells in the presence of a chemotherapeutic agent, is the key to successful therapy. Thus, the most appropriate chemotherapeutic agent for this kind of treatment is not the usual appropriate drug for each specific situation but a non-permeant drug (such as bleomycin) for which the cytotoxicity will be highly potentiated by tumour cell electropermeabilization, whatever the carcinological situation.

ECT has been demonstrated to be an effective local treatment that can be added to the usual set of local anti-cancer therapies. For the treatment of metastatic diseases it is important to add a systemic component to ECT. Therefore, in preclinical trials, ECT has been combined with immunotherapy. Systemic effect demonstrated by responses of distant tumours was obtained by combining local ECT with the local administration of histoincompatible interleukin 2 (IL-2)-secreting cells in a murine model (Mir et al, 1995). In addition, local anti-tumour effect was enhanced by the local administration of IL-2 (Mir et al, 1992). Thus, the combination of ECT with immunotherapy may lead to a wider applicability of the technique by producing a systemic anti-tumour treatment.

New possibilities of ECT are presently being explored in preclinical trials with the use of cisplatin. Although cisplatin is a more permeant drug than bleomycin its effectiveness was also augmented by electropermeabilization of cells in vitro as well as tumours in vivo (Serša et al, 1995). Cisplatin is currently used in many chemotherapeutic protocols. Therefore, ECT with cisplatin can be useful in patients as an adjuvant to ongoing cisplatin treatment. These results are encouraging and suggest that the effectiveness of other anti-tumour agents may also be augmented by electropermeabilization.

### ACKNOWLEDGEMENTS

We acknowledge Alice Fidon for her secretarial help, and all our colleagues. Work of the authors was supported by grants from Centre National de la Recherche Scientifique (CNRS), Institut Gustave-Roussy (Contrat de Recherche 92-14), Institut Electricite Santé (IES) and Association pour le développement de la Recherche sur le Cancer (ARC) (LMM, SO and JB), CNRS, ARC, IES and Fondation pour la Recherche Médicale (JT and MPR), Ministry of Science and Technology of the Republic of Slovenia (GS and DM) and Genetronics, San Diego, CA, USA and University of South Florida Departments of Surgery and Chemical Engineering (RH and RG).

### REFERENCES

Belehradek J Jr, Orlowski S, Poddevin B, Paoletti C and Mir LM (1991) Electrochemotherapy of spontaneous mammary tumors in mice. *Eur J Cancer* **27**: 73–76

Belehradek M, Domenge C, Luboinski B, Orlowski S, Belehradek J Jr and Mir LM (1993) Electrochemotherapy, a new antitumor treatment: first clinical phase I–II trial. *Cancer* **72**: 3694–3700

Belehradek J Jr, Orlowski S, Ramirez LH, Pron G, Poddevin B and Mir LM (1994) Electropermeabilization of cells in tissues assessed by the qualitative and quantitative electroloading of bleomycin. *Biochim Biophys Acta* **1190**: 155–163

Buzaid AC and Murren J (1992) Chemotherapy for advanced malignant melanoma. *Int J Clin Lab Res* **21**: 205–209

Čemažar M, Miklavčič D, Vodovnik L, Jarm T, Zvonimir R, Štabuc B, Cufer T and Serša G (1995) Improved therapeutic effect of electrochemotherapy with cisplatin by intratumoral drug administration and changing of electrode orientation for electropermeabilization on EAT tumor model in mice. *Radiol Oncol* **29**: 121–127

Coates AS (1992) Systemic chemotherapy for malignant melanoma. *World J Surg* **16**: 277–278

Domenge C, Orlowski S, Luboinski B, De Baere T, Schwaab G, Belehradek J Jr and Mir LM (1996) Antitumor electrochemotherapy: new advances in the clinical protocol. *Cancer* **77**: 956–963

Heller R (1995) Treatment of cutaneous nodules using electrochemotherapy. *J FL Med Assoc* **82**: 147–150

Heller R, Jaroszeski M, Leo-Messina J, Perrot R, Van Voorhis N, Reintgen D and Gilbert R (1995) Treatment of B16 mouse melanoma with the combination of electropermeabilization and chemotherapy. *Bioelectrochem Bioenerg* **36**: 83–87

Heller R, Jaroszeski MJ, Glass LF, Messina JL, Rapaport DP, Fenske NA, Gilbert R, Mir LM and Reintgen DS (1996) Phase I/II trial for the treatment of cutaneous and subcutaneous tumors using electrochemotherapy. *Cancer* **77**: 964–971

Mir LM (1994) L'électrochimiothérapie antitumorale. *Bull Cancer* **81**: 740–748

Mir LM, Banoun H and Paoletti C (1988) Introduction of definite amounts of nonpermeant molecules into living cells after electropermeabilization: direct access to the cytosol. *Exp Cell Res* **175**: 15–25

Mir LM, Orlowski S, Belehradek J Jr and Paoletti C (1991) Electrochemotherapy: potentiation of antitumor effect of bleomycin by local electric pulses. *Eur J Cancer* **27**: 68–72

Mir LM, Orlowski S, Poddevin B and Belehradek J Jr (1992) Electrochemotherapy tumor treatment is improved by interleukin-2 stimulation of the host's defenses. *Eur Cytokine Netw* **3**: 331–334

Mir LM, Roth C, Orlowski S, Quintin-Colonna F, Fradelizi D, Belehradek J Jr and Kourilsky P (1995) Systemic antitumor effects of electrochemotherapy combined with histoincompatible cells secreting interleukin-2. *J Immunother* **17**: 30–38

Mir LM, Tounekti O and Orlowski S (1996) Bleomycin: revival of an old drug. *Gen Pharmacol* **27**: 745–748

Nathanson L and Jilani S (1993) Chemotherapy of malignant melanoma. *Cancer Treat Rev* **19**: 17–28

Neumann E, Sowers AE and Jordan CA (1989) *Electroporation and Electrofusion in Cell Biology*. Plenum Press: New York

Orlowski S and Mir LM (1993) Cell electropermeabilization: a new tool for biochemical and pharmacological studies. *Biochim Biophys Acta* **1154**: 51–63

Orlowski S, Belehradek J Jr, Paoletti C and Mir LM (1988) Transient electropermeabilization of cells in culture: increase of the cytotoxicity of anticancer drugs. *Biochem Pharmacol* **37**: 4727–4733

Poddevin B, Orlowski S, Belehradek J Jr and Mir LM (1991) Very high cytotoxicity of bleomycin introduced into the cytosol of cells in culture. *Biochem Pharmacol* **42**(suppl.): 67–75

Pron G, Belehradek J Jr and Mir LM (1993) Identification of a plasma membrane protein that specifically binds bleomycin. *Biochem Biophys Res Comm* **194**: 333–337

Pron G, Belehradek J Jr, Orlowski S and Mir LM (1994) Involvement of membrane bleomycin-binding sites in bleomycin cytotoxicity. *Biochem Pharmacol* **48**: 301–310

Rols MP and Teissié J (1990) Electropermeabilization of mammalian cells: a quantitative analysis of the phenomenon. *Biophys J* **58**: 1089–1098

Rudolf Z, Štabuc B, Čemažar M, Miklavčič D, Vodovnik L and Serša G (1995) Electrochemotherapy with bleomycin. The first clinical experience in malignant melanoma patients. *Radiol Oncol* **29**: 229–235

Salford LG, Persson BRR, Brun A, Ceberg CP, Kongstad PC and Mir LM (1993) A new brain tumor therapy combining bleomycin with in vivo electropermeabilization. *Biochem Biophys Res Comm* **194**: 938–943

© Cancer Research Campaign 1998

Serša G, Čemažar M, Miklavčič D and Mir LM (1994) Electrochemotherapy: variable anti-tumor effect on different tumor models. *Bioelectrochem Bioenerg* **35**: 23–27

Serša G, Čemažar M and Miklavčič D (1995) Antitumor effectiveness of electrochemotherapy with cis-diamminedichloroplatinum (II) in mice. *Cancer Res* **55**: 3450–3455

Tounekti O, Pron G, Belehradek J Jr and Mir LM (1993) Bleomycin, an apoptosis-mimetic drug that induces two types of cell death depending on the number of molecules internalized. *Cancer Res* **53**: 5462–5469

Yamaguchi O, Irisawa C, Baba K, Ogihara M, Yokota T and Shiraiwa Y (1994) Potentiation of antitumor effect of bleomycin by local electric pulses in mouse bladder tumor. *Tohoku J Exp Med* **172**: 291–293

Yeung RS (1994) Management of recurrent cutaneous melanoma. *Curr Probl Cancer* **18**: 143–186

*© Cancer Research Campaign 1998*


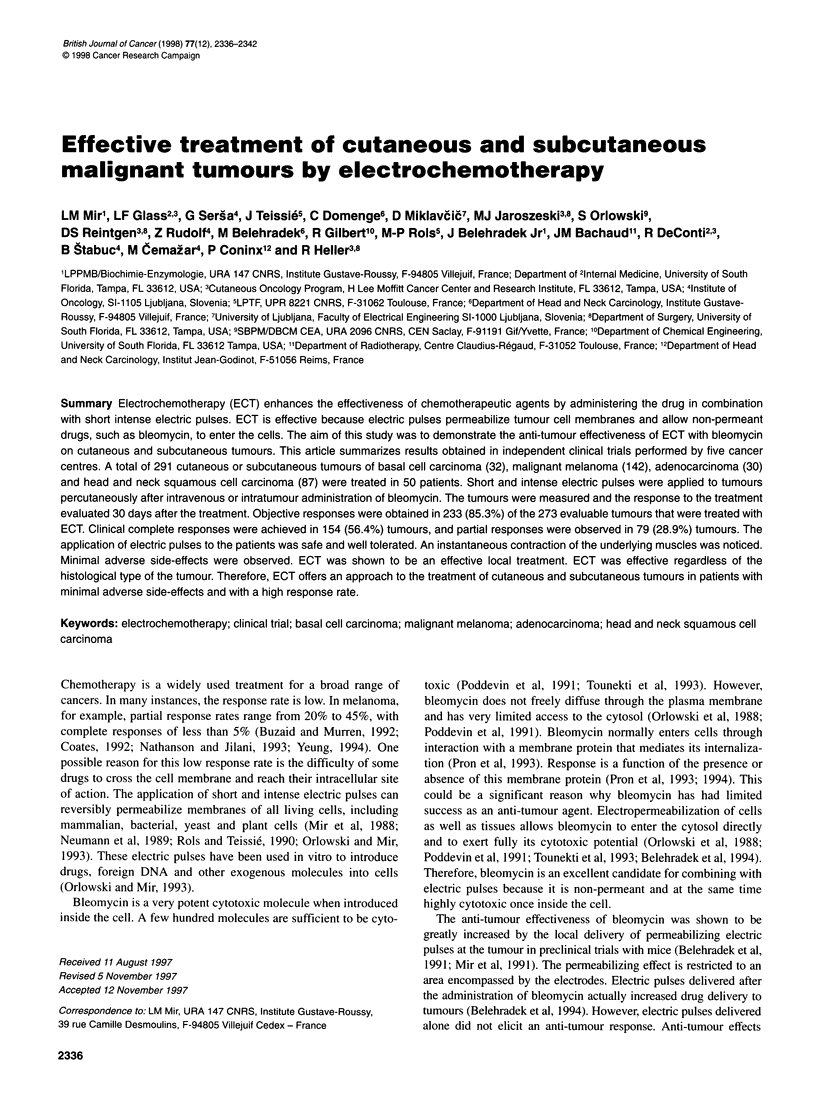

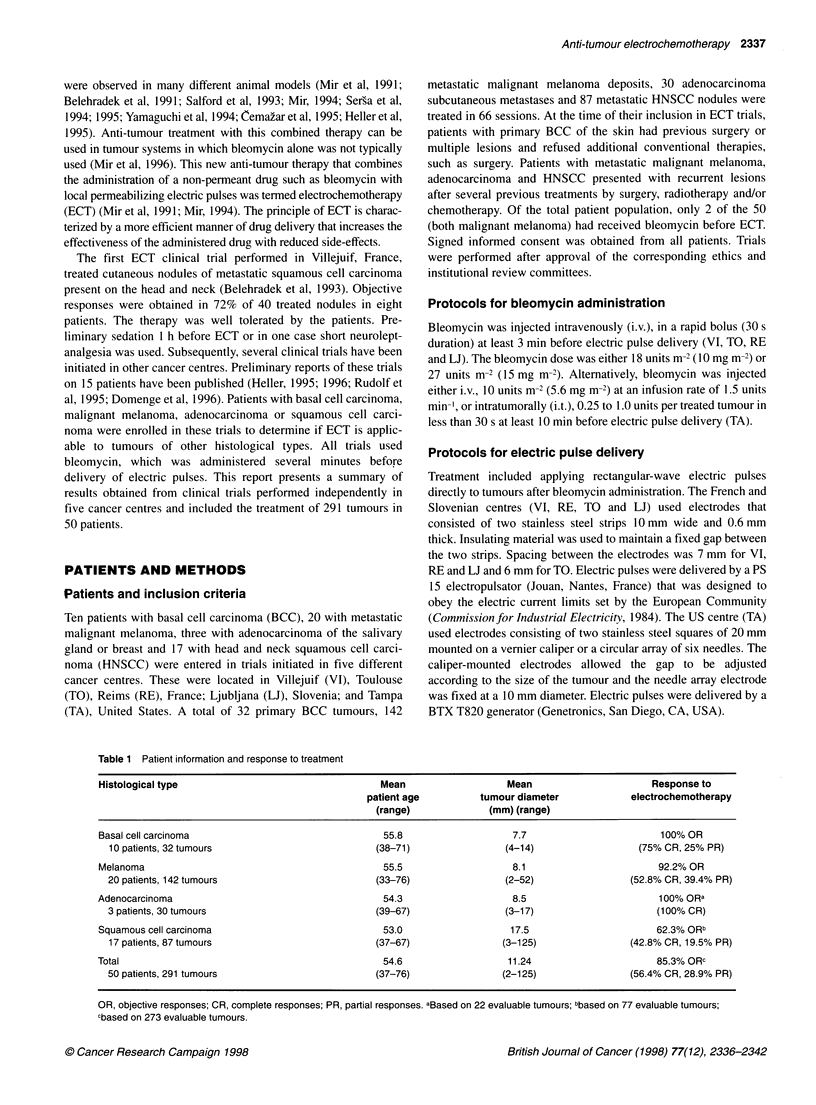

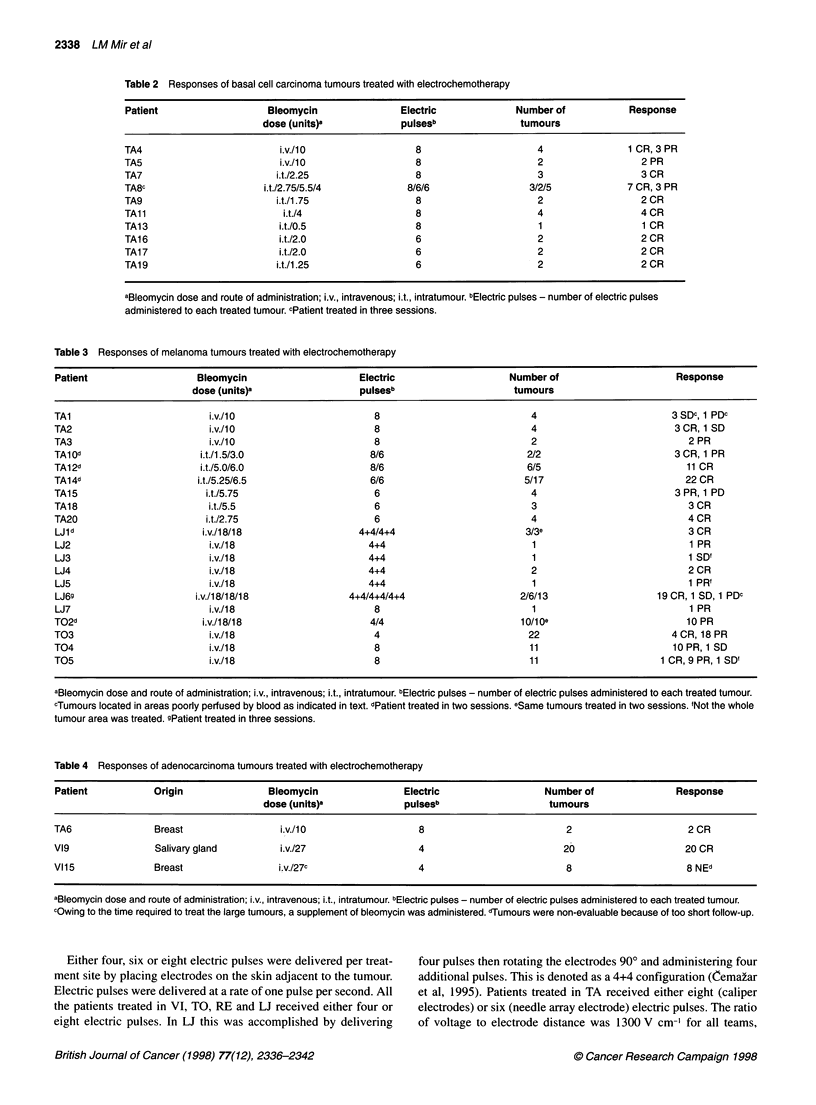

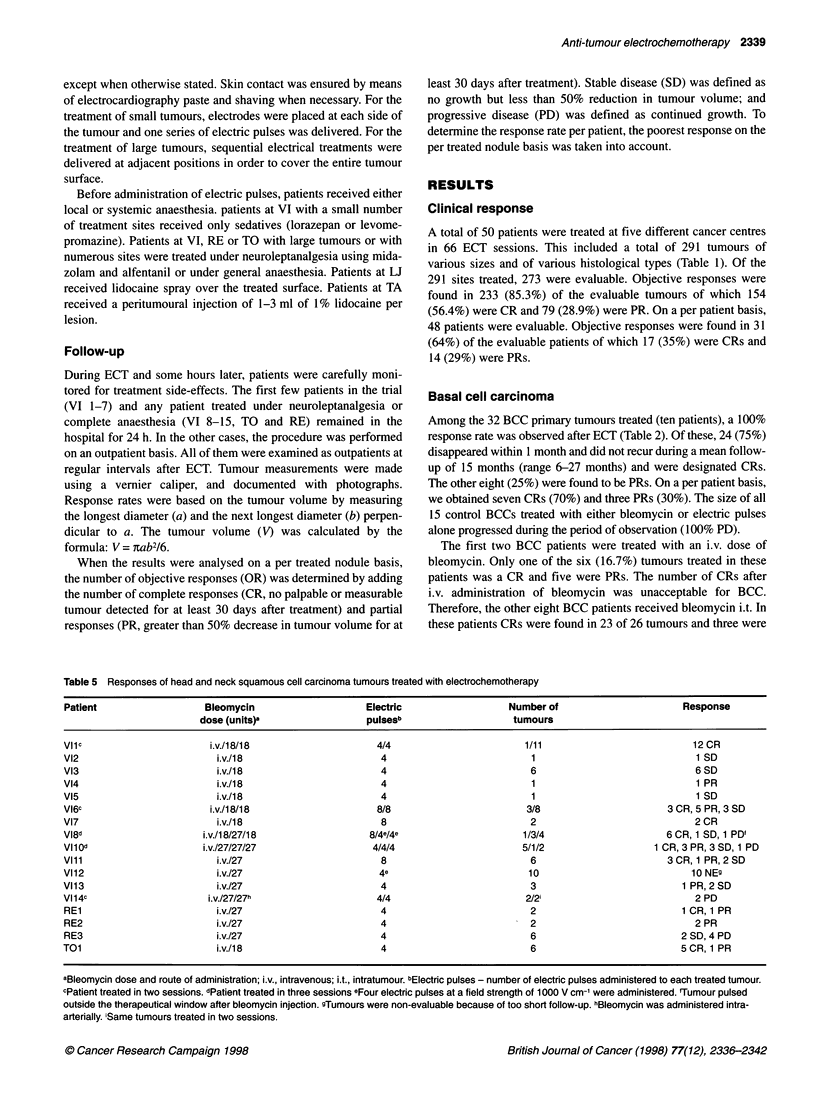

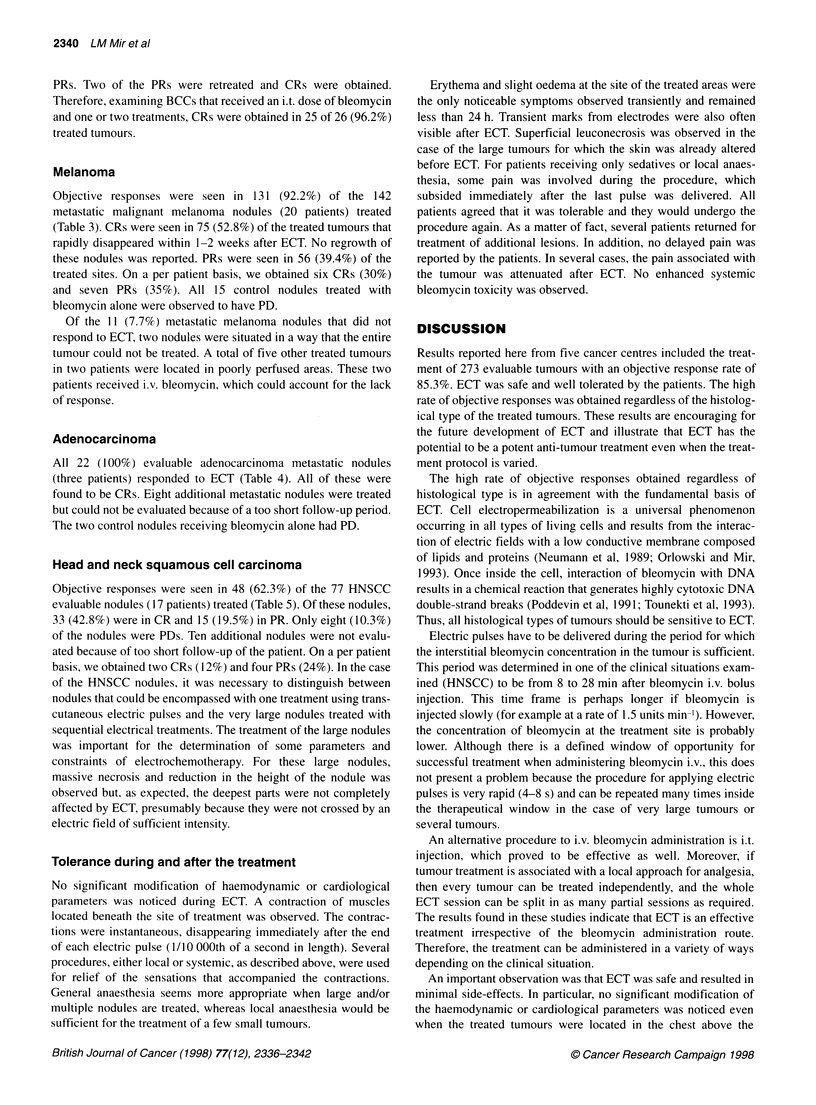

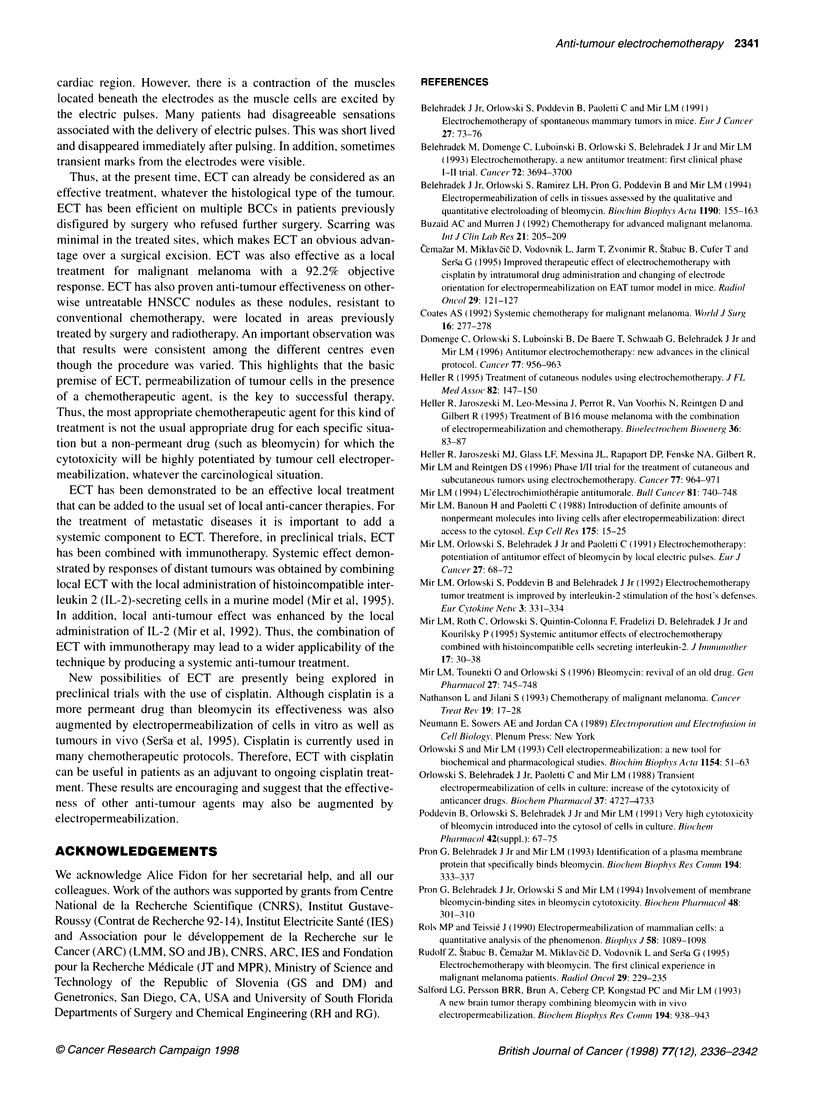

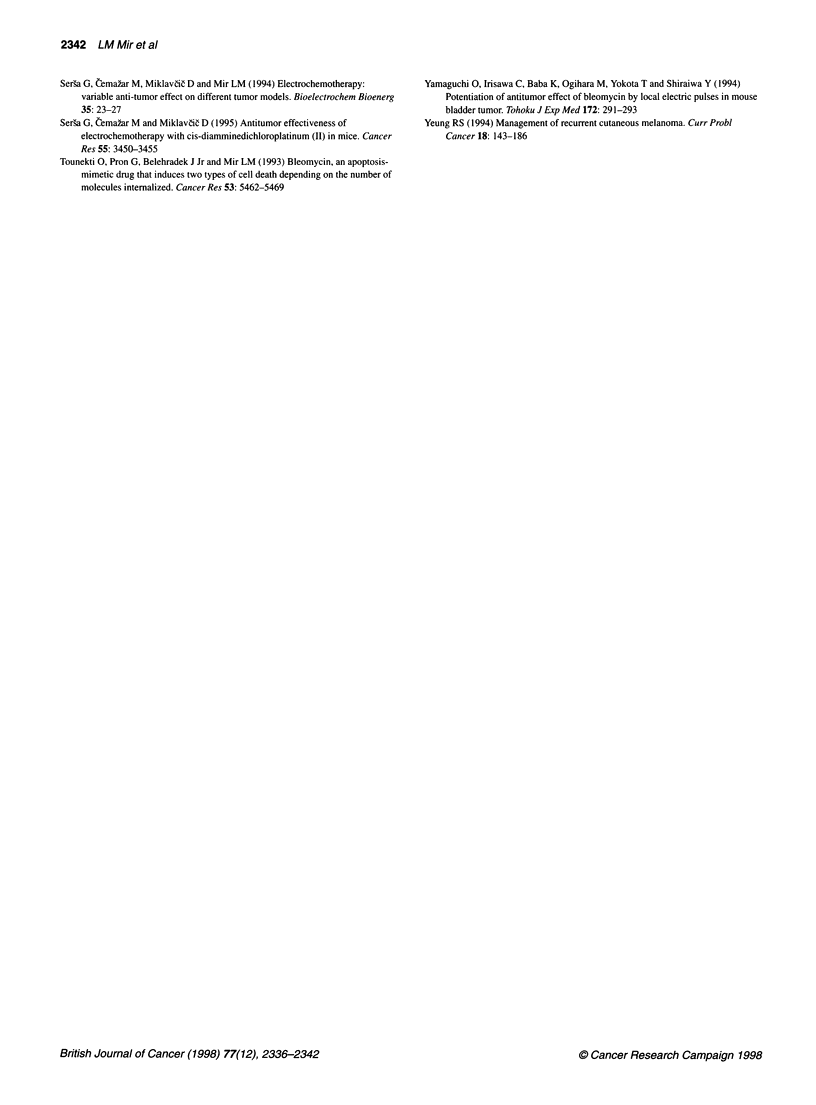


## References

[OCR_00625] Belehradek J., Orlowski S., Poddevin B., Paoletti C., Mir L. M. (1991). Electrochemotherapy of spontaneous mammary tumours in mice.. Eur J Cancer.

[OCR_00635] Belehradek J., Orlowski S., Ramirez L. H., Pron G., Poddevin B., Mir L. M. (1994). Electropermeabilization of cells in tissues assessed by the qualitative and quantitative electroloading of bleomycin.. Biochim Biophys Acta.

[OCR_00628] Belehradek M., Domenge C., Luboinski B., Orlowski S., Belehradek J., Mir L. M. (1993). Electrochemotherapy, a new antitumor treatment. First clinical phase I-II trial.. Cancer.

[OCR_00638] Buzaid A. C., Murren J. (1992). Chemotherapy for advanced malignant melanoma.. Int J Clin Lab Res.

[OCR_00650] Coates A. S. (1992). Systemic chemotherapy for malignant melanoma.. World J Surg.

[OCR_00656] Domenge C., Orlowski S., Luboinski B., De Baere T., Schwaab G., Belehradek J., Mir L. M. (1996). Antitumor electrochemotherapy: new advances in the clinical protocol.. Cancer.

[OCR_00670] Heller R., Jaroszeski M. J., Glass L. F., Messina J. L., Rapaport D. P., DeConti R. C., Fenske N. A., Gilbert R. A., Mir L. M., Reintgen D. S. (1996). Phase I/II trial for the treatment of cutaneous and subcutaneous tumors using electrochemotherapy.. Cancer.

[OCR_00659] Heller R. (1995). Treatment of cutaneous nodules using electrochemotherapy.. J Fla Med Assoc.

[OCR_00675] Mir L. M., Banoun H., Paoletti C. (1988). Introduction of definite amounts of nonpermeant molecules into living cells after electropermeabilization: direct access to the cytosol.. Exp Cell Res.

[OCR_00674] Mir L. M. (1994). L'électrochimiothérapie antitumorale.. Bull Cancer.

[OCR_00680] Mir L. M., Orlowski S., Belehradek J., Paoletti C. (1991). Electrochemotherapy potentiation of antitumour effect of bleomycin by local electric pulses.. Eur J Cancer.

[OCR_00685] Mir L. M., Orlowski S., Poddevin B., Belehradek J. (1992). Electrochemotherapy tumor treatment is improved by interleukin-2 stimulation of the host's defenses.. Eur Cytokine Netw.

[OCR_00690] Mir L. M., Roth C., Orlowski S., Quintin-Colonna F., Fradelizi D., Belehradek J., Kourilsky P. (1995). Systemic antitumor effects of electrochemotherapy combined with histoincompatible cells secreting interleukin-2.. J Immunother Emphasis Tumor Immunol.

[OCR_00697] Mir L. M., Tounekti O., Orlowski S. (1996). Bleomycin: revival of an old drug.. Gen Pharmacol.

[OCR_00712] Orlowski S., Belehradek J., Paoletti C., Mir L. M. (1988). Transient electropermeabilization of cells in culture. Increase of the cytotoxicity of anticancer drugs.. Biochem Pharmacol.

[OCR_00709] Orlowski S., Mir L. M. (1993). Cell electropermeabilization: a new tool for biochemical and pharmacological studies.. Biochim Biophys Acta.

[OCR_00722] Pron G., Belehradek J., Mir L. M. (1993). Identification of a plasma membrane protein that specifically binds bleomycin.. Biochem Biophys Res Commun.

[OCR_00727] Pron G., Belehradek J., Orlowski S., Mir L. M. (1994). Involvement of membrane bleomycin-binding sites in bleomycin cytotoxicity.. Biochem Pharmacol.

[OCR_00734] Rols M. P., Teissié J. (1990). Electropermeabilization of mammalian cells. Quantitative analysis of the phenomenon.. Biophys J.

[OCR_00741] Salford L. G., Persson B. R., Brun A., Ceberg C. P., Kongstad P. C., Mir L. M. (1993). A new brain tumour therapy combining bleomycin with in vivo electropermeabilization.. Biochem Biophys Res Commun.

[OCR_00756] Sersa G., Cemazar M., Miklavcic D. (1995). Antitumor effectiveness of electrochemotherapy with cis-diamminedichloroplatinum(II) in mice.. Cancer Res.

[OCR_00761] Tounekti O., Pron G., Belehradek J., Mir L. M. (1993). Bleomycin, an apoptosis-mimetic drug that induces two types of cell death depending on the number of molecules internalized.. Cancer Res.

[OCR_00766] Yamaguchi O., Irisawa C., Baba K., Ogihara M., Yokota T., Shiraiwa Y. (1994). Potentiation of antitumor effect of bleomycin by local electric pulses in mouse bladder tumor.. Tohoku J Exp Med.

[OCR_00771] Yeung R. S. (1994). Management of recurrent cutaneous melanoma.. Curr Probl Cancer.

